# Hard Enough to Manage My Emotions: How Hardiness Moderates the Relationship Between Emotional Demands and Exhaustion

**DOI:** 10.3389/fpsyg.2020.01194

**Published:** 2020-06-18

**Authors:** Greta Mazzetti, Dina Guglielmi, Gabriela Topa

**Affiliations:** ^1^Department of Education Studies, University of Bologna, Bologna, Italy; ^2^Department of Psychology, Universidad Nacional de Educación a Distancia, Madrid, Spain

**Keywords:** emotional demands, conflict, hardiness, emotional exhaustion, health care sector, nurses, Job Demands–Resources (JD-R) model

## Abstract

The frequency of conflicts with patients’ families is one of the main contributors to the amount of emotional demands that healthcare professionals must tackle to prevent the occurrence of burnout symptoms. On the other hand, research evidence suggests that hardiness could enable healthcare professionals to handle their responsibilities and problems effectively. Based on the health impairment process of the Job Demands–Resources model, the main goal of this study was to delve deeper into the relationship between conflict with patients’ families, emotional demands, and exhaustion, as well as to test the buffering role of hardiness. Data were collected from a sample of *N* = 295 healthcare professionals working in a private hospital in Northern Italy. Most of them were women (78.6%) with a mean age of 40.62 years (SD = 9.50). The mediation of emotional demands within the association between conflict with families and emotional exhaustion and the moderating role of hardiness was tested using a bootstrapping approach. In the current sample, emotional demands mediated the association between conflict with families and exhaustion among healthcare professionals. Moreover, this relationship decreased among individuals with higher levels of hardiness. These findings contribute to the current understanding of the negative impact played by conflict with families on healthcare professionals’ psychological well-being. Furthermore, they corroborated the role of hardiness as a personal resource that could prevent the occurrence of burnout symptoms. In addition to manage—and decrease—episodes of conflict with patients and their families, organizations in the healthcare sector should develop interventions aimed at fostering employees’ hardiness and, consequently, tackle job demands ingrained in their profession (i.e., emotional demands).

## Introduction

Healthcare professionals represent one of the occupational populations most frequently involved in studies aimed at exploring the incidence of emotional exhaustion (e.g., Van Mol et al., 2015). This evidence could be explained by the work environment and the demands faced by these professionals. Healthcare professionals must take on considerable responsibilities concerning patients’ health and well-being to tackle traumatic and painful life events that affect their patients’ lives and to cope with the emotional demands stemming from these situations (Riley and Weiss, 2016). A significant portion of the emotional demands perceived among healthcare professionals could arise from the constant interaction with patients’ families. For instance, they are required to make difficult decisions in critical medical situations and to discuss them with families using suited communication strategies and attention to their emotional needs (Vicent et al., 2019). These situations may lead to conflictual interaction with patients’ relatives that, in turn, may negatively impact the quality of care provided, jeopardize the cohesion of healthcare work teams and have a detrimental impact on professionals’ well-being (Fassier and Azoulay, 2010). Accordingly, the current study draws on the assumption that the establishment of an emotional bond with families facing a stressful life event, such as assisting ill relatives, could imply the occurrence of conflicts between families and healthcare professionals.

These conflictual relationships may arise, for instance, from disagreements about patients’ treatment, communication breakdown, and unrealistic expectations concerning the outcome of care. They could contribute to the amount of emotional demands that physicians and nurses perceive in their daily activities (Forbat et al., 2016). This emotional overload may, in turn, exhaust employees’ mental and physical resources and, therefore, translate into a higher occurrence of burnout symptoms (i.e., emotional exhaustion). Drawing on the health-impairment process postulated by the Job Demands–Resources model (JD-R model; Schaufeli and Taris, 2014), this study was aimed to clarify the mechanism through which conflict with patients’ families contributes to emotional exhaustion by proposing emotional demands as a transmitting variable. Moreover, we posit that this process is conditional. In other words, the indirect association between emotional demands and exhaustion is expected to be buffered by higher levels of hardiness, which is hypothesized to act as a protective factor of psychological well-being among healthcare professionals.

### The Role of Emotions in the Job Demands–Resources Model

During the last decades, one of the leading models in the job stress literature has been the JD-R model (Schaufeli and Taris, 2014), which postulates the presence of two underlying psychological processes that trigger the development of workers’ strain and motivation. On the one hand, the health-impairment process posits that a prolonged exposure to aspects of work entailing a significant expenditure of energy (i.e., job demands) may deplete workers’ psychological, emotional, and physical resources. This progressive depletion flows into a condition of emotional exhaustion that represents the central component of burnout and the first dimension to develop, subsequently eliciting symptoms of cynicism and reduced personal efficacy (Maslach et al., 2001). Emotional exhaustion is characterized by persistent feelings of tiredness and a shortage of energy in executing one’s daily work tasks (Mäkikangas and Kinnunen, 2016). As reported in a noteworthy review of burnout literature (Patel et al., 2018), emotional exhaustion is related to a wide range of adverse work-related (e.g., impaired job performance, higher turnover rates), and individual outcomes (e.g., substance use, depressive symptoms).

On the other hand, the motivational process of the JD-R model postulates that the availability of psychological and social features enabling workers to achieve work goals (i.e., job resources) may foster employees’ motivation. According to this framework, adequate job resources may enhance work engagement (Schaufeli and Taris, 2014), defined as a positive psychological state characterized by the combination of vigor (i.e., the willingness to invest higher energy at work and to persist even in the face of difficulties), dedication (i.e., feelings of passion and involvement toward one’s job), and absorption (i.e., feelings of being immersed in one’s job). This condition can promote positive organizational and individual outcomes, such as improved job performance and workers’ well-being (Guglielmi et al., 2013). A core characteristic of the JD-R model is its flexibility. In other words, this overarching theoretical framework can be applied to any occupational setting, regardless of the particular demands and resources involved. Still, it also can be tailored to select the distinctive risk factors and protective features (job demands and job resources, respectively) able to trigger and prevent job stress conditions in each specific occupational sector (Bakker and Demerouti, 2017).

The current study focused on the occurrence of conflicts with patients’ families and the subsequent perception of emotional demands, since these job demands are crucial among professionals working in the healthcare sector, especially regarding emotional exhaustion. The management of conflict with patients’ relatives requires investing a considerable quantity of staff time, mainly when they stem from communication complexities due to cross-cultural differences (Brierley et al., 2013).

These conflictual situations entail direct and indirect costs at the organizational level, such as the undermining of team morale, decreased trust between healthcare staff (i.e., nurses and physicians) and patients’ relatives, and the possibility of legal proceedings (Forbat et al., 2016).

The main goal for the healthcare professional is to deliver high-quality care that safeguards patient safety but also to contain costs. Accordingly, demanding contacts with relatives are considered as a significant source of emotional demands among healthcare professionals, who are also expected to perform efficiently when facing patients suffering and to cope with aggressive patients’ behavior (Sundin et al., 2008). Burnout literature indicates that the effort performed by workers in their attempt to manage their feelings properly could represent a major cause of intense physiological fatigue, thus triggering increased levels of emotional exhaustion (Simbula et al., 2019). Based on this rationale, the following hypothesis was tested:

**Hypothesis 1.** *Emotional demands mediate the relationship between conflict with patients’ families and emotional exhaustion. Thus, the occurrence of conflicts with families is positively related to emotional demands, which in turn are associated with higher levels of exhaustion among healthcare professionals.*

### The Buffering Role of Hardiness

According to the motivational process of the JD-R model (Schaufeli, 2014), job resources have a motivational potential both at the intrinsic (i.e., by stimulating employees’ learning and development) and extrinsic level (i.e., by allowing employees to achieve their goals). Additionally, the buffering hypothesis proposed by the JD-R model restates that job resources have the potential to decrease the harmful association between job demands and strain—in this study, emotional exhaustion (Bakker and Demerouti, 2014). There is compelling evidence from the JD-R model literature suggesting that, in addition to job resources, also personal resources could act as significant protective factors for workers’ well-being. Personal resources are defined as positive cognitions and self-evaluation regarding employees’ ability to manage effectively and influence their environment (Xanthopoulou et al., 2009). For instance, previous findings suggest that proactive personality acts as a protecting factor able to hinder the onset of conflicts with clients in the service sector, particularly among workers experiencing high levels of emotional dissonance (Mazzetti et al., 2019). Accordingly, the current study was aimed to investigate the role of hardiness as a personal resource able to reduce the detrimental association between emotional demands and symptoms of emotional exhaustion among healthcare professionals.

The construct of hardiness—or hardy personality—was developed by Kobasa (1979) to describe a personality structure built upon the inclination to derive meaning from stressful events, the perception of control on life events, and the tendency to experience any occurring change as an opportunity to learn and grow. Hence, hardiness originates from the combination of three key dimensions: commitment to life and work, sense of control over the events, and the aptitude to experience changes as challenges. *Commitment* is characterized by experiencing one’s job as intrinsically stimulating; *control* is defined as perceiving oneself as able to influence life events; and *challenge* describes the tendency to interpret changes as natural events that allow developing new abilities (Maddi, 2008). In line with this definition, hardiness can be conceived as a personal resource that protects healthcare professionals facing demanding situations and enables them to implement adaptive behaviors. By reframing demands as challenges toward which they can exert adequate control, these workers are enabled to implement effective coping strategies (Delahaij et al., 2010). This assumption is supported by the evidence that hardiness is negatively related to burnout symptoms, in particular, the depletion of cognitive and emotional energy—emotional exhaustion—and cynism (Alarcon et al., 2009; Adriaenssens et al., 2015). Accordingly, the current study was aimed to contribute to the literature on hardiness by exploring the buffering role of this personal resource in the indirect association between conflict with patients’ families and emotional exhaustion. Based on the related theory and empirical evidence summarized, the second study hypothesis was formulated as follows:

**Hypothesis 2.** *Within the association between conflict with patients’ families, emotional demands, and exhaustion, hardiness can reduce the relationship between emotional demands and exhaustion. Hence, we expect that healthcare professionals characterized by greater hardiness also display poorer symptoms of exhaustion in comparison to their colleagues reporting a lower level of hardiness.*

**FIGURE 1:**
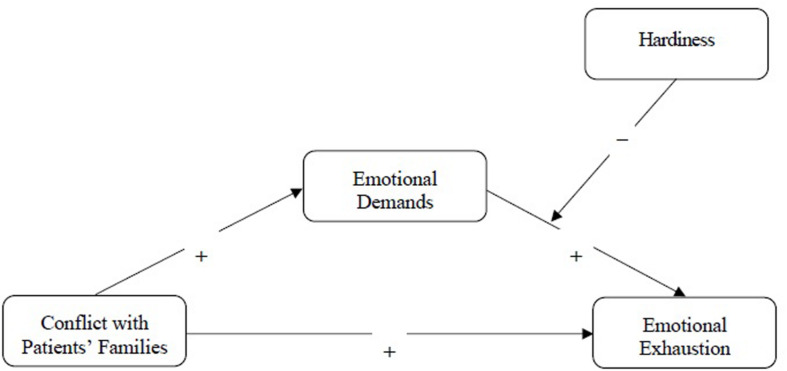
The hypothesized moderated mediation model. Control variables (gender and age) are omitted for reasons of clarity.

The hypothesized moderated mediation is depicted in Figure 1.

## Materials and Methods

### Participants and Procedures

The current study was part of a project on occupational health and psychosocial risk assessment in a private hospital located in Northern Italy. In particular, healthcare professionals working in this context participated in a short presentation session held by four members of the University research group that was aimed to present the general aims of the project. The head physicians of each ward were asked to distribute the paper-and-pencil questionnaire to their staff. The survey included a cover letter reporting background information about the general aim of the study, detailed information regarding data processing, and a description of the measures implemented to prevent unauthorized access to personal data. On the first page of the questionnaire, participant anonymity was emphasized, and confidentiality guaranteed, following the guidelines for personal data treatment defined by the General Data Protection Regulation (GDPR) and Italian privacy law (Law Decree DL-196/2003 and at. 89 of EU REGULATION 2016/679). This introduction also stated that participation in this study was voluntary and that informed consent was implied through survey completion. Participants were asked to place the filled-out questionnaires in a ballot box located in each hospital ward. A total of 295 healthcare professionals returned the completed questionnaire. Concerning their professional role, the majority of the sample consisted of nurses (28.3%), physiotherapists (25.2%), medical assistants (21.3%), physicians (12.8%), and intermediate care technicians (12.4%). Most participants were women (78.6%), and the age ranged from 23 to 64 years old, with a mean age of 40.62 years (SD = 9.50). Among them, 41.5% had an organizational tenure between 11 and 20 years, 21.8% reported an organizational tenure between 6 and 10 years, 17.7% between 1 and 5 years, and the remaining 19% of the sample has been working in the current hospital for more than 20 years.

### Measures

#### Conflict With Patients’ Families

The frequency of unfriendly interactions and disagreements about protocols, treatment aims, and inadequate communication with patients’ families was assessed with the three-item scale adapted from the survey validated by Guglielmi et al. (2011).

Sample items were: “I am in conflict with the patients’ family members” and “Interacting with patients’ family members is frustrating.” Participants were asked to rate how often they experienced the situation described using a 5-point frequency scale from 1 = *never* to 5 = *very often.* Thus, greater scores indicated a higher occurrence of conflictual interactions with patients’ families. In the present study, the reliability of the scale was α = 0.83.

#### Emotional Demands

The emotional load stemming from daily activities carried out by healthcare professionals, such as performing painful procedures and communicating unpleasant news to patients, was measured using four items taken from the Nursing Stress Scale (Gray-Toft and Anderson, 1981). Sample items are: “How often in your job do you experience the death of a patient with whom you developed a close relationship?” and “How often do you feel helpless in the case of a patient who fails to improve?” Each item was rated on a Likert scale ranging from 1 = *never* to 4 = *very frequently.* The internal consistency of the scale yielded a Cronbach’s alpha of α = 0.75.

#### Hardiness

This personal resource was assessed through the Occupational Hardiness Questionnaire validated by Moreno-Jiménez et al. (2014). The survey includes three subscales covering the core dimensions of hardiness. Each subscale consists of five items: challenge (e.g., “In my job, I feel attracted to tasks and situations involving a personal challenge”), control (e.g., “The control of situations is the only thing that ensures success”), and commitment (e.g., “My own excitement is what makes me go ahead with the completion of my activity”). The response options varied on a 4-point agreement scale ranging from 1 = *completely disagree* to 4 = *completely agree*. The internal consistency of this scale was α = 0.85.

#### Emotional Exhaustion

The core dimension of burnout was assessed using the corresponding subscale of the Maslach Burnout Inventory (Schaufeli et al., 1996). The scale included five items, with higher scores indicating a greater occurrence of symptoms of exhaustion. Sample items are: “I feel emotionally drained by my work” and “I feel fatigued when I wake up in the morning and have to face another day on the job.” Participants were asked to assess each item on a frequency scale ranging from 0 = *never* to 6 = *every day*. In this study, the Cronbach’s alpha was set at α = 0.84.

### Strategy of Analysis

The hypotheses were tested using the Process macro for SPSS (Hayes, 2013). To be specific, the mediating effect of emotional demands in the relationship between conflict with patients’ families and emotional exhaustion (*Hypothesis* 1) was tested using Model 4. Furthermore, Model 14 was performed to assess the moderated mediation model (*Hypothesis 2*) postulating that conflict with patients’ families (i.e., the independent variable) is associated with higher levels of emotional demands (i.e., the mediating variable). Emotional demands, in turn, are related to higher levels of emotional exhaustion (i.e., the criterion variable), and this association is expected to be moderated by participants’ hardiness (i.e., the moderator).

These models were tested using 5,000 bootstrap samples in order to obtain reliable estimates of standard errors and confidence intervals. Gender and age were included as covariates, given their association with emotional demands and exhaustion (Purvanova and Muros, 2010; Brienza and Bobocel, 2017).

## Results

### Descriptive Results

Descriptive statistics for all study variables are reported in Table 1. It should be noted that all significant associations between the constructs under investigation were in the expected direction. As displayed on the diagonal of this table, all scales have satisfactory reliabilities, with Cronbach’s alpha coefficients higher than 0.70.

**TABLE 1:** Mean, SD, and correlation among study variables (*N* = 295).

	***M***	**SD**	***r***					
			**1**	**2**	**3**	**4**	**5**	**6**
1. Gender (1 = male)	0.21	0.41	−					
2. Age	40.61	9.50	0.23***	−				
3. Conflict with patients’ families	2.42	0.93	–0.10	−0.24***	*(0.83)*			
4. Emotional demands	2.98	0.85	−0.13*	−0.12*	0.33***	*(0.75)*		
5. Hardiness	3.08	0.44	0.06	–0.01	−0.16**	–0.10	*(0.85)*	
6. Emotional exhaustion	3.24	1.46	−0.20**	−0.25***	−0.42***	0.35***	−0.21***	*(0.84)*

*p < 0.05; **p < 0.01; ***p < 0.001; and Cronbach’s alpha in brackets along the diagonal.

### Testing the Mediating Role of Emotional Demands

The assumption that emotional demands mediate the relationship between conflict with patients’ families and emotional exhaustion (*Hypothesis 1*) was tested using bootstrap analysis. The obtained results indicated a significant direct relationship between conflict with patients’ families and emotional exhaustion: *b* (*SE*) = 0.50 (0.09), *p* = 0.000, and 95% CI (0.33;0.67).

Furthermore, conflict with patients’ families reported a significant association with emotional demands: *b* (*SE*) = 0.27 (0.05), *p* = 0.000, and 95% CI (0.17;0.37). Emotional demands, in turn, were positively related to emotional exhaustion: *b* (*SE*) = 0.40 (0.09), *p* = 0.000, and 95% CI (0.21;0.58). The estimated indirect relationship between conflict with patients’ families and exhaustion *via* emotional demands was statistically significant: *b* (*SE*) = 0.11 (0.03), and 95% CI (0.05;0.18). Approximately 27% of the variance in emotional exhaustion was accounted for by the predictors (*R*^2^ = 0.268). These results supported the mediating role of emotional demands in explaining the relationship between the frequency of conflicts with families and the occurrence of symptoms referred to emotional exhaustion. This evidence provided support to *Hypothesis 1.*

### Testing the Moderated Mediation Model

Table 2 displays the results of the hypothesized moderated mediation model (*Hypothesis 2*). The mediating model (to emotional demands) revealed that conflict with patients’ families was significantly associated with a higher perception of emotional demands [*b* = 0.27, *SE* = 0.05, *p* = 0.000, and 95% CI (0.17;0.37)]. The dependent variable model indicated that a higher perception of emotional demands was positively associated with symptoms of emotional exhaustion [*b* = 0.36, *SE* = 0.09, *p* = 0.000, and 95% CI (0.18;0.55)]. Taken together, these results indicate that a greater frequency of conflictual relationships with patients’ families was related to higher emotional demands which, in turn, reported a positive association with the occurrence of exhaustion symptoms. Furthermore, hardiness reported a negative association with emotional exhaustion [*b* = −0.40, *SE* = 0.16, *p* = 0.017, and 95% CI (−0.73; −0.07)]. The interaction between emotional demands and hardiness on exhaustion was significant [*b* = −0.57, *SE* = 0.17, *p* = 0.001, and 95% CI (−0.92; −0.22)], as well as the index of moderated mediation [*b* = −0.15, *SE* = 0.06, and 95% CI (−0.28; −0.06)].

**TABLE 2:** Results of the moderated mediation model for emotional exhaustion.

**Variable**	**Emotional demands (*M*)**		**Emotional exhaustion (*Y*)**	
	***Coefficient***	***SE***	***Coefficient***	***SE***
Gender (1 = male)	–0.21	0.11	−0.37*	0.18
Age	–0.01	0.01	−0.02*	0.01
Conflict with patients’ families (*X*)	0.27**	0.05	0.46**	0.08
Emotional demands (*M*)			0.36**	0.09
Hardiness (*W*)			−0.40*	0.16
Emotional demands *X* Hardiness			−0.57**	0.17

Model of *M* Summary	*R*^2^ = 0.12**			

Model of *Y* Summary			*R*^2^ = 0.31**	

***Conditional indirect effect of conflict with patients’ families (X) on emotional exhaustion (Y) through emotional demands (M) at values of hardiness (W)***				

**Hardiness**	***Effect***	***Boot SE***	***Boot 95% CI***	

*Lower levels*	0.61	0.12	0.38;0.85	
*Middle levels*	0.36	0.09	0.18;0.55	
*Higher levels*	0.11	0.13	−0.13;0.36	

*N* = 295; *p < 0.05; and **p < 0.01.

**FIGURE 2:**
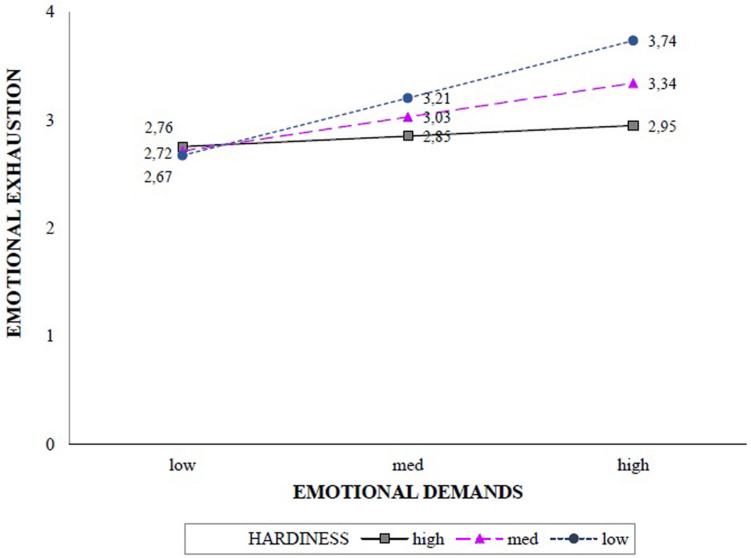
Interaction effect of emotional demands and hardiness in predicting emotional exhaustion.

According to the values of the moderator reported in the lower part of Table 2, the indirect relationship between conflict with patients’ families and emotional exhaustion through emotional demands was significant at low [−1 *SD*; *b* = 0.61, *SE* = 0.12, *p* = 0.000, and 95% CI (0.38;0.85)] and medium [*mean*; *b* = 0.36, *SE* = 0.09, *p* = 0.000, and 95% CI (0.18;0.55)] levels of hardiness. In contrast, this indirect association was not significant at high levels of hardiness [+1 *SD*; *b* = 0.11, *SE* = 0.13, *p* = 0.368, and 95% CI (−0.13;0.36)]. This interaction effect is displayed in Figure 2. Healthcare professionals characterized by low and medium levels of hardiness reported higher symptoms of emotional exhaustion. Overall, these results supported *Hypothesis 2.*

Among the control variables considered in the current model, higher levels of emotional exhaustion were reported by women (*b* = -0.37, *p* = 0.040), and younger participants (*b* = −0.02, *p* = 0.011).

## Discussion

This study was aimed to delve deeper into the process that links the occurrence of conflicts with patients’ families and the core dimension of job burnout—,i.e., emotional exhaustion—through the transmitting role played by emotional demands using a sample of healthcare professionals. A second goal was to empirically assess the moderating role of hardiness as a personal resource that could reduce the harmful relationship between emotional demands and symptoms of exhaustion. The obtained results provided support to the assumption that higher levels of exhaustion may result from a greater occurrence of dysfunctional relationships with patients’ relatives and the subsequent burden of emotionally demanding tasks. In line with previous results, emotional demands represent a category of job demands particularly crucial in the healthcare sector. They entail the requirement to perform efficiently even when facing situations particularly intense in emotional terms: interacting with suffering patients and families, dealing with the dramatic consequences of unsuccessful procedures, and containing strong emotional reactions (Van Keer et al., 2015; Riedl and Thomas, 2019). The mediating model tested in the current research corroborated the assumption that the relationship between conflictual relationships with families and the frequency of exhaustion symptoms can be explained through the amplified amount of emotional demands. According to these findings, the chronic exposure to unceasing requests from patients and their families can be greatly expensive in terms of resources invested by workers, thus are likely to drain their emotional and cognitive energies (Blanco-Donoso et al., 2016; Aiello and Tesi, 2017).

The current research also supports the protective role of professionals’ hardiness against the negative outcome of the health-impairment process described in the JD-R model. In line with previous findings, this study indicates that hardiness can reduce the harmful association between the emotionally demanding aspects of healthcare professions and the symptoms of exhaustion. In other words, hardy nurses, physicians, and care technicians can persevere with commitment and a sense of control in their daily work activities, even in the face of heavy emotional demands (Mazzetti et al., 2019).

On the other hand, the current study has some limitations that should be mentioned. The main weakness comes from the adoption of a cross-sectional design that prevents from drawing indisputable conclusions on the causal link among the study variables. Future studies based on longitudinal data could evaluate the occurrence of reciprocal causal relationships between emotional exhaustion and the perception of higher job demands (i.e., conflict with patients’ families and emotional demands). Furthermore, the current study postulated a theoretical model that conceived emotional demands as a predictor of exhaustion, in line with the health-impairment process of the JD-R model. Nonetheless, it did not consider the direction of their association with work engagement. Previous evidence suggests that emotional demands could represent a challenging feature of healthcare professions that can foster nurses’ creativity and motivation toward their work (De Jonge et al., 2008; Eley et al., 2012).

An additional limitation of the current research is related to the nature of our sample, which consisted of healthcare professionals working in a single private hospital. Therefore, an interesting venue for future research would be to replicate the current model in a different working population that is likely to experience conflictual relationships with customers and clients, such as employees from the service sector. This extension would allow to corroborate the current results and evaluate whether they are generalizable across different contexts.

These present findings also provide relevant indications for the opportunity to train healthcare professionals in order to help them in managing the emotional demands of their work. To this purpose, training activities could foster strategies for emotional labor. In particular, deep acting strategies are based on the effort of adjusting the perception of emotional events to genuinely experience the emotions required by one’s work role or context (Gabriel and Diefendorff, 2015). This antecedent-focused strategy allows eliciting the required emotion and, consequently, it could protect one’s sense of authenticity. The enactment of deep acting strategies could prevent the state of emotional dissonance stemming from exhibiting unauthentic emotions to fit with the requirements, particularly in terms of display rules, prescribed by one’s role (Hobfoll et al., 2018). Although deep acting strategies require the investment of a considerable amount of resources, they could prevent the occurrence of strain symptoms that originate by the constant effort to display unauthentic emotions (Hülsheger and Schewe, 2011). A further venue entails training paths aimed to foster workers’ hardiness, which is defined as a malleable characteristic that could be developed through dedicated initiatives (Sudom et al., 2014). This strategy would be in line with the motivating potential of personal resources depicted by the JD-R model. In essence, a higher level of hardiness could be expected to stimulate workers’ growth and development (Schaufeli and Salanova, 2010).

A further implication entails the promotion of an approach that leads to experience one’s job as more engaging and meaningful (i.e., job crafting strategies). As a result, workers can proactively tackle their job demands, prevent the occurrence of counterproductive work behavior and perceive higher levels of well-being (Ingusci et al., 2016; Guglielmi et al., 2017). Previous findings among nurses’ managers revealed that hardiness training could boost workers’ confidence in their ability to influence the course of events and to translate demanding situations into learning opportunities (Judkins et al., 2006). According to these findings, hardiness training for the entire staff can boost workers’ resources and, from a broader perspective, craft a healthy work environment where professionals perform their duties efficiently and experience a condition of intrinsic motivation and satisfaction.

## Data Availability Statement

The data are available upon request to the first author (greta.mazzetti3@unibo.it).

## Ethics Statement

Ethical review and approval was not required for the study on human participants in accordance with the local legislation and institutional requirements. Written informed consent for participation was not required for this study in accordance with the national legislation and the institutional requirements.

## Author Contributions

DG and GM contributed to the conceptualization. GM contributed to the formal analysis. GM and DG contributed to the investigation and writing. GM contributed to the methodology. DG and GT contributed to the supervision. GM and DG contributed to the writing of the original draft. GT contributed to the review and editing. All authors contributed to the manuscript and approved the submitted version.

## Conflict of Interest

The authors declare that the research was conducted in the absence of any commercial or financial relationships that could be construed as a potential conflict of interest.
